# Continuous therapy response references for *BCR::ABL1* monitoring in pediatric chronic myeloid leukemia

**DOI:** 10.1038/s41598-023-45364-0

**Published:** 2023-10-24

**Authors:** Christian Volz, Thomas Zerjatke, Andrea Gottschalk, Sabine Semper, Meinolf Suttorp, Ingmar Glauche, Manuela Krumbholz, Markus Metzler

**Affiliations:** 1 Department of Pediatrics and Adolescent Medicine, University Hospital Erlangen, Erlangen, Germany; 2 Friedrich-Alexander-Universität Erlangen-Nürnberg, Erlangen, Germany; 3 Faculty of Medicine Carl Gustav Carus, Institute for Medical Informatics and Biometry, Technische Universität Dresden, Dresden, Germany; 4 Pediatric Hemato-Oncology, Faculty of Medicine Carl Gustav Carus, Technische Universität Dresden, Dresden, Germany; 5 Comprehensive Cancer Center Erlangen-EMN (CCC ER-EMN), Erlangen, Germany; 6 Bavarian Cancer Research Center (BZKF), Erlangen, Germany

**Keywords:** Haematological cancer, Paediatric research

## Abstract

Response to tyrosine kinase inhibitor (TKI) therapy in patients with chronic myeloid leukemia (CML) is monitored by quantification of *BCR::ABL1* transcript levels. Milestones for assessing optimal treatment response have been defined in adult CML patients and are applied to children and adolescents although it is questionable whether transferability to pediatric patients is appropriate regarding genetic and clinical differences. Therefore, we analyzed the molecular response kinetics to TKI therapy in 129 pediatric CML patients and investigated whether response assessment based on continuous references can support an early individual therapy adjustment. We applied a moving quantiles approach to establish a high-resolution response target curve and contrasted the median responses in all patients with the median of the ideal target curve obtained from a subgroup of optimal responders. The high-resolution response target curve of the *optimal responder group* presents a valuable tool for continuous therapy monitoring of individual pediatric CML patients in addition to the fixed milestones. By further comparing *BCR::ABL1* transcript levels with *BCR::ABL1* fusion gene copy numbers, it is also possible to model the differential dynamics of *BCR::ABL1* expression and cell number under therapy. The developed methodology can be transferred to other biomarkers for continuous therapy monitoring.

## Introduction

Tyrosine kinase inhibitors (TKI) are effective target-specific inhibitors of the constitutively active *BCR::ABL1* oncoprotein characterizing chronic myeloid leukemia (CML). The introduction of the first-generation TKI imatinib has distinctly improved treatment response, progression-free survival, and overall survival of patients with CML^1–4^. For many years, imatinib was the only TKI approved for the treatment of children. With the approval of second-generation TKIs (2G-TKI) dasatinib and nilotinib as first-line therapy in children and adolescents, there are now additional options to switch to potent alternatives^5–7^ and sustained treatment-free remission (TFR) emerged as a realistic therapy aim for a substantial number of adults and pediatric CML patients^8–13^. Therefore, achieving TFR through stringently controlled therapy is more important today, particularly for young patients to avoid side effects such as growth retardation and potential long-term effects of lifelong treatment^4,6,13–15^. Consequentially, it is desirable to achieve deep molecular response (DMR) as early as possible, potentially before the end of the growth phase.

It is currently unknown, whether 2G-TKIs may have more or less adverse effects on pediatric bone metabolism^16,17^. Therefore, imatinib continues to be recommended as a first-line therapy in children, due to its generally good efficacy and the largest clinical experience. In case of a non-optimal response, rapid switching to a 2G-TKI is the strategy for achieving a faster DMR.

Therapy assessment is based on the National Comprehensive Cancer Network guidelines and the European LeukemiaNet (ELN), which are derived from study data in adult CML patients^18,19^. Besides the hematologic and cytogenetic response, the focus is on the molecular response (MR) as measured by the *BCR::ABL1* transcript level from peripheral blood cells according to the International Scale (IS)^19^. The ELN defines therapy milestones based on the quantification of MR at months 3 (*BCR::ABL1* ≤ 10%), 6 (*BCR::ABL1* ≤ 1%), and 12 (*BCR::ABL1* ≤ 0.1%) and categorizes response into optimal, warning, and failure^19^. However, while residual disease at month 6 is a strong predictor of achieving MR and DMR, month 3 seems to be less informative for children. Children often present with very high leukocyte count and pronounced organomegaly at diagnosis such the early milestones are frequently not reached under imatinib treatment^20–24^. For improved treatment monitoring, a high-resolution response target curve based on pediatric-specific reference values would be necessary, especially within the first 6 months after treatment initiation.

Therefore, we developed a quantitative reference model based on 2372 pediatric *BCR::ABL1* transcript measurements for continuous assessment of therapy response in children and adolescents. We compared the overall course in all patients with the course in an *optimal responder group* (patients with an optimal response at month 6 and 12). Further focusing on the *optimal responder group,* we established a reference curve to continuously monitor the response of pediatric patients. For the first time, this approach allows for a pediatric-specific evaluation of therapy response based on a quantitative assessment of *BCR::ABL1* levels beyond the fixed milestones at months 3, 6, and 12. This is particularly important in children where continuous therapy response monitoring is required to optimize TKI dosing, to monitor compliance, or to assist with temporary or permanent TKI cessations for the reduction of side effects.

To further complement this monitoring approach, we measured DNA-based *BCR::ABL1* fusion gene and RNA-based transcript levels in parallel in 64 pediatric CML patients during their treatment. Since the abundance of *BCR::ABL1* fusion gene correlates better with CML cell number as compared to *BCR::ABL1* transcript levels, we apply a non-linear mixed effect model to analyze the ratio of these two observables to achieve a better understanding of how much *BCR::ABL1* expression changes throughout treatment.

## Materials and methods

### Patients

Treatment response to first-line imatinib was analyzed from 129 pediatric CML patients diagnosed in the chronic phase enrolled in the CML-PAED-II trial (NCT00445822, EudraCT 2007-001339-69) and the subsequent registry (2007 to 2022) (Table 1). Both were conducted in accordance with the Declaration of Helsinki and were approved by institutional ethics review boards of the medical faculties of the Technical University, Dresden, Germany and the Friedrich-Alexander-Universität Erlangen-Nürnberg (FAU), Germany (EC282122006 and EC 236_18B). Informed consent was obtained from patients and/or legal guardians.

**Table 1:** Characteristics of the whole patient cohort and optimal responder group, respectively.

		Whole study cohort (n = 129 patients)	*Optimal responder group* (n = 43 patients)
Age at diagnosis [y]		Median 14 (1–18)	Median 14 (4–17)
Sex	Male	n = 79 (61%)	n = 31 (72%)
	Female	n = 50 (39%)	n = 12 (28%)
Transcript type	e13a2	n = 48	n = 14
	e14a2	n = 56	n = 21
	e13a2/e14a2	n = 9	n = 2
	Unknown	n = 16	n = 6
Therapy	First-line	ima (n = 129)	ima (n = 43)
	Second-line	ima to dasa (n = 48)	ima to dasa (n = 10)
		ima to nilo (n = 2)	ima to nilo (n = 0)
	Third-line	dasa to nilo (n = 9)	dasa to nilo (n = 2)
		dasa to pona (n = 1)	dasa to pona (n = 0)
		dasa to bosu (n = 1)	dasa to bosu (n = 0)
TKI discontinuation		n = 13 (10%)	n = 11 (26%)
Time in TFR [mo]		Median 4 (3–12) [4 patients ongoing]	Median 6 (3–12) [4 patients ongoing]

*y* years, *ima* imatinib, *dasa* dasatinib, *nilo* nilotinib, *pona* ponatinib, *bosu* bosutinib, *TKI* tyrosine kinase inhibitor, *TFR* treatment-free remission, *mo* months.

For each patient, the time from therapy start to first reaching MR3 (ratio *BCR::ABL1/ABL1* ≤ 0.1% IS) was calculated. The cumulative probability was derived by a Kaplan–Meier analysis that was stratified by patient age or sex, respectively. The correlation of initial leukocyte numbers and achievement of milestones at month 3 and 6 was analyzed using a U test statistic. Logistic regression was performed on the log-transformed initial leukocyte counts to estimate the risk of not achieving these milestones.

### *BCR::ABL1* transcript and fusion gene levels

Transcript levels were normalized to the IS with a detection limit of MR4.5. The quantification of patient‐specific fusion gene levels was performed in 64/129 patients as described previously^25^ using a digital droplet PCR assay with a detection limit of at least MR4.5. RNA and DNA levels were compared with GraphPad prism software (v9.0.2)^26^ using Spearman correlation statistics.

### Moving median, moving quantiles analyses

A moving median approach was used to obtain a smoothed time course for the average treatment response in the cohort to be analyzed. For each time point t_i_ we calculated the median *BCR::ABL1* transcript level for all patients with available measurements in a specified time interval of 3 months centered around t_i_. If multiple measurements were available for the same patient, we first calculated a patient-specific mean in order to not bias the median approach extending over all patients. We varied the time point t_i_ between diagnosis (t_i_ = 0) and 3 years of follow-up (t_i_ = 36 m) as long as measurements were available at the late time points. The resulting curve was smoothed with a LOESS regression.

The same approach was taken for the calculation of the 25%-, 75%- and 95%-quantiles. Analyses and visualization were performed with the statistical programming environment R (v4.2.1)^27^.

### Biexponential model

We used a bi-exponential parameterization of the *BCR::ABL1* transcript and fusion gene level time courses to obtain characteristics of the treatment dynamics. All data were normalized to the first measurement. We describe the time courses on the log scale using the logarithm of a bi-exponential function, i.e. log(*BCR::ABL1*) = log(*A* exp(α * t) + B exp(β * t)), with t being the time measured in months after therapy start. Each patient’s response dynamic is therefore defined by the parameters A, B, α, and β. These parameters are estimated for all patients by fitting the bi-exponential model to the patients’ *BCR::ABL1* transcript levels using a population-based non-linear mixed effect model (NLME) as described before for adult patients^28^. For undetectable measurements, a detection limit of MR4.5 was used as a left-censored value. For fitting and evaluating the NLME model, we applied the software Monolix (v2021R1)^29^. RNA- and DNA-based monitoring parameters were compared using paired t-tests. Analyses and visualization besides Monolix were performed using Mathematica 13.1^30^.

## Results

### Patient characteristics

**Figure 1:**
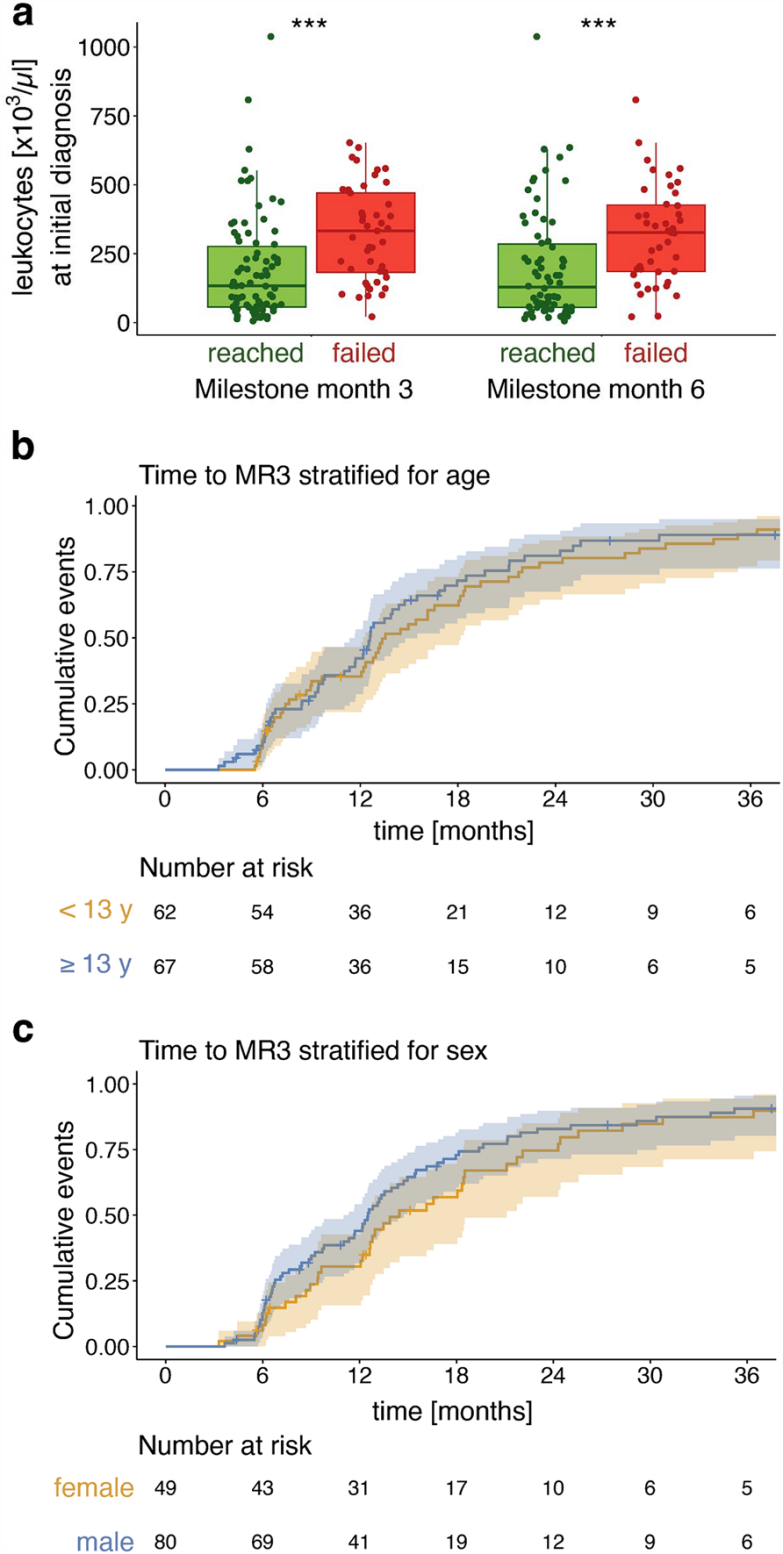
Prognostic influence of patient characteristics and initial leukocyte count. (**a**) Leukocyte count at diagnosis in pediatric CML patients who reach ELN milestones at months 3 and 6 compared to those who miss them. ***p < 0.001 (U test) (**b**) Time in months to reach MR3 stratified for age (cut off ≥ 13 years) and sex (**c**). y years.

The median leukocyte count in our cohort of 129 pediatric CML patients at the time of diagnosis was 189.2 × 10^3^/µl (range: 5.6–1037 × 10^3^/µl), significantly higher than typical median numbers between 60 and 80 × 10^3^/µl described for adult patients^31,32^. Analyzing the impact of highly elevated leukocyte counts on treatment response, we found that patients failing to reach the milestones at months 3 and 6 had on average significantly higher initial leukocyte counts (mean initial leukocyte counts at month 3 323.6 × 10^3^/µl vs. 191.7 × 10^3^/µl, at month 6 320.1 × 10^3^/µl vs. 199.7 × 10^3^/µl) (Fig. 1a). Estimating the increasing risk of missing milestones 3 or 6 by a logistic regression revealed odds ratios of 10.3 or 7.3, respectively, for each tenfold increase in the initial leukocyte count (Fig. S1).

A time-to-MR3-based risk stratification considering the age at diagnosis and sex shows no age-dependent effect but male patients reach MR3 slightly earlier, however not significantly (Fig. 1b,c).

### Moving quantiles analysis in pediatric CML

**Figure 2:**
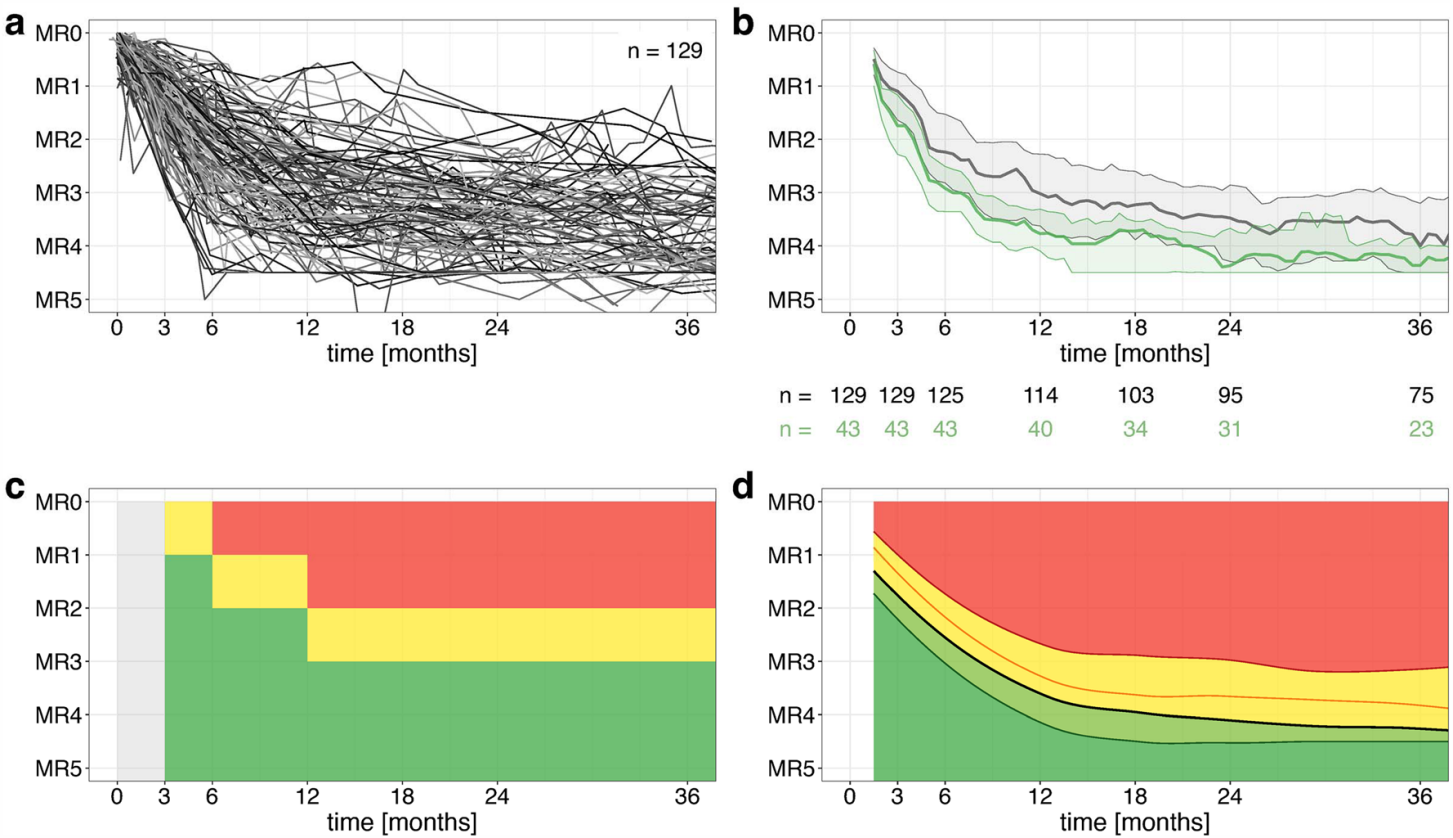
Moving quantiles analyses for calculation of continuous references for pediatric-specific *BCR::ABL1* transcript monitoring. (**a**) Monitoring of individual *BCR::ABL1* transcript levels of all patients during TKI. (**b**) Moving quantiles based on *BCR::ABL1* transcript numbers of all patients (gray) or *optimal responders* (green) plotted over the first 3 years of TKI therapy. The dark-colored lines represent the median (50-Q.). Light-colored lines present the 25-Q. and the 75-Q. (**c**) Visualization of ELN criteria over the first 3 years of TKI therapy. The green area marks optimal therapy response, the yellow area indicates warning and the red area represents therapy failure. (**d**) Continuous references based on moving quantiles analyses of *optimal responders* over the first 3 years of TKI therapy. The smoothed graphs illustrate the 25-Q. (green line), 50-Q. (grey line), 75-Q. (orange line) and 95-Q. (red line). The light and dark green areas represent an above-average MR (optimal response), the yellow area a slightly below-average MR (observation range), and the red area a non-optimal MR (poor response). Q. quantile.

Analyses were performed based on 2372 *BCR::ABL1* transcript values from 129 pediatric patients. The individual patient courses during TKI treatment over 36 months are shown in Fig. 2a. We applied a moving quantiles approach to obtain an average response for all patients as well as for a subgroup of 43 optimal responders. The *optimal responder group* included patients reaching the ELN milestones in month 6 and 12, patients with missing *BCR::ABL1* transcript data at months 6 or 12 (± 30 days) were excluded. Clinical characteristics of this patient cohort are listed in Table 1.

Figure 2b compares the 50%-quantiles (50-Q; median molecular response) over the first 3 years of therapy assessment between the whole cohort (gray) and the *optimal responder group* (green). The MR shows a biphasic dynamic with a steeper decrease in the first 12 months and a slower decrease thereafter. There is a marked downward shift in the distribution of quantiles in the *optimal responder group*. The median of all patients maps closest to the ELN milestones indicating that approximately half of all pediatric CML patients were not adjusted to the optimal therapy range. The median of the *optimal responder group* achieves a 0.6, 0.7, and 0.8 log deeper MR at months 3, 6, and 12, respectively (Table 2).

**Table 2:** Molecular response (MR) at month 3, 6 and 12 and the time span in months to reach MR3 of the 25%-, 50%-, 75%- and 95%- quantiles (25-Q., 50-Q., 75-Q., 95-Q.) from the moving quantiles analysis for the entire study cohort (all patients) and for the optimal responder group compared to the ELN milestones.

		MR at month 3	MR at month 6	MR at month 12	Time to MR3 [m]
All patients cohort (n = 129 pts)	25-Q	MR1.6	MR2.8	MR3.6	8.1
	50-Q	MR1.1	MR2.2	MR3.0	13.0
	75-Q	MR0.7	MR1.5	MR2.3	21.1
	95-Q	MR0.4	MR0.8	MR1.4	43.9
*Optimal responder cohort* (n = 43 pts)	*25-Q*	MR2.3	MR3.4	MR4.1	6.0
	*50-Q*	MR1.7	MR2.9	MR3.8	6.6
	*75-Q*	MR1.2	MR2.5	MR3.3	9.8
	*95-Q*	MR0.7	MR2.2	MR3.1	12.5
ELN milestones		MR1	MR2	MR3	

*pts* patients, *m* months.

According to ELN criteria, there are fixed milestones at months 3, 6, and 12 to assess therapy response (Fig. 2c). We suggest complementing this rigid approach with a continuous, milestone-independent perspective, in which average time courses of *optimal responders* are taken as the reference frame. Figure 2d presents the smoothed curve (= high-resolution response target curve) of the *optimal responder group* curve shown in Fig. 2b. In addition to the 25%-, 50%-, and 75%-quantiles, the 95%- quantile is depicted.

### Continuous therapy response references for *BCR::ABL1* MR monitoring in pediatric CML patients

**Figure 3:**
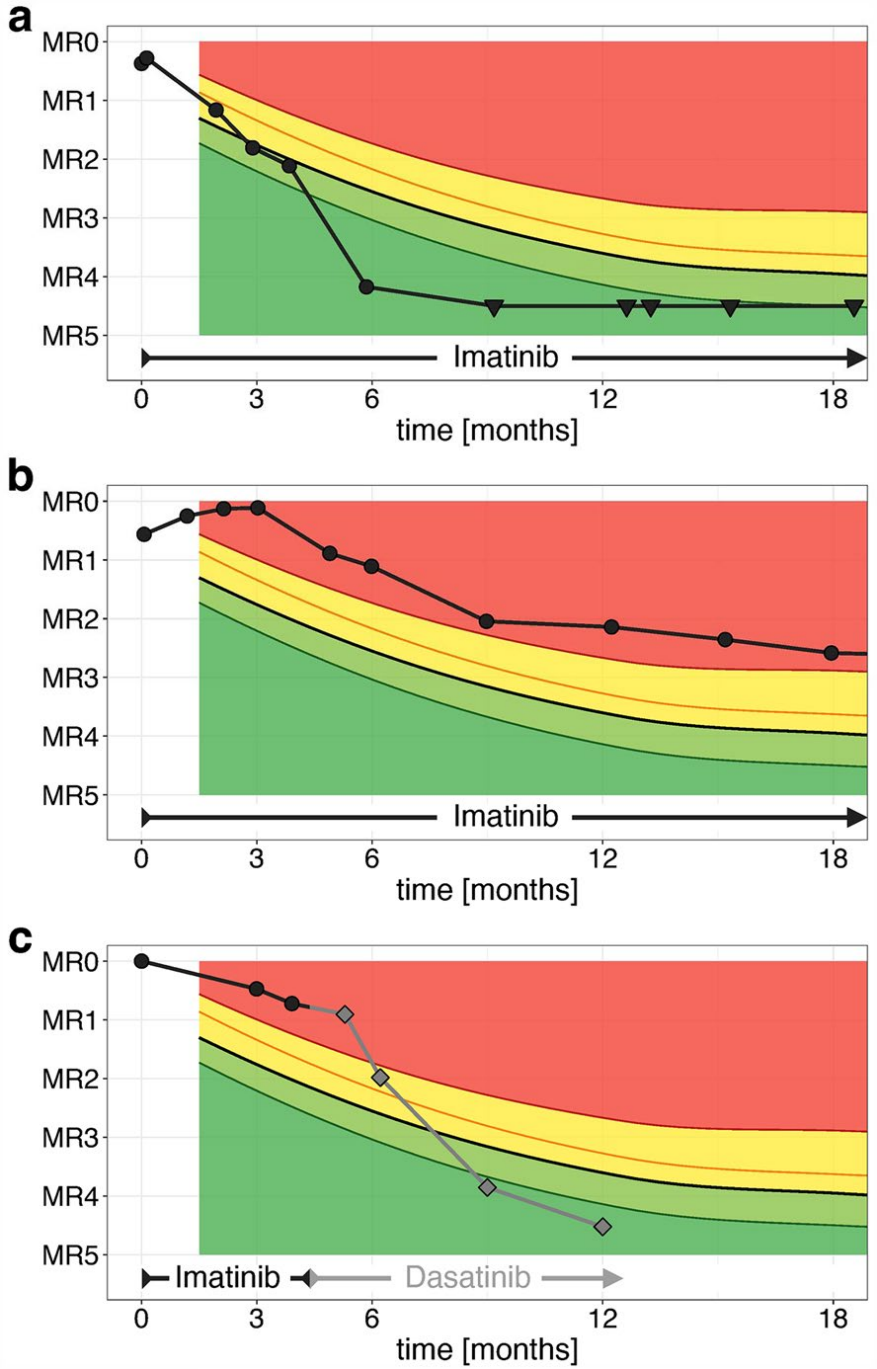
Monitoring patients’ individual therapy response by plotting their MR course over 18 months against the continuous references of the optimal responders. (**a**) a male patient (4 years old at diagnosis, e14a2 transcript) with uninterrupted imatinib therapy (**b**) a female patient (15 years old at diagnosis, e13a2 + e14a2 transcript) with uninterrupted imatinib therapy, and (**c**) a male patient (13 years old at diagnosis, e13a2 transcript) with imatinib therapy for 131 days and a subsequent TKI switch to dasatinib. Black circles represent measurements under the imatinib treatment; grey squares represent measurements under the dasatinib treatment. Triangles indicate non-detectable *BCR::ABL1* transcript levels.

Plotting the individual MR course against the moving quantiles (Fig. 2d), every *BCR::ABL1* value at any time point can be evaluated and assessed relative to the high-resolution response target curve. MR values within the green range (below 50-Q.), exemplarily shown in the patient course of Fig. 3a, can be rated as a desirable MR. In contrast, *BCR::ABL1* transcript levels in the red range must be classified as poor MR (Fig. 3b). The yellow area between 50-Q. and 95-Q. represents an observation (warning) range.

With the ability to assess all measurements non-optimal treatment responders can be monitored more closely and a dose adaption or TKI switching can be initiated at an early stage as exemplarily shown in Fig. 3c. Initially starting with a 100% *BCR::ABL1* transcript level, the *BCR::ABL1* transcripts decreased insufficiently (33.63%) during the first three months with imatinib treatment. At month 4, *BCR::ABL1* transcripts further decreased (18.93%) but still inadequately. With two *BCR::ABL1* values in the non-optimal red range, a switch to the 2G-TKI dasatinib was performed at month 4.5 (1.5 months before ELN milestone 6), whereupon *BCR::ABL1* transcripts decreased substantially and the patient reached the optimal MR range at the follow-up month 9 (0.014%) and 12 (0.003%).

### *BCR::ABL1* transcript expression in CML cells during imatinib treatment

DNA-based detection of the *BCR::ABL1* fusion gene appears as a more sensitive measure of residual CML, especially in deep remission^25,33,34^. Therefore, it might be beneficial to monitor RNA and DNA in parallel to decide on TKI treatment cessation and the potential for achieving TFR. The *BCR::ABL1* fusion gene level correlates closely with the absolute CML cell numbers, while the *BCR::ABL1* transcript level depends on the transcriptional activity. Therefore, the RNA/DNA ratio can be used as an estimator of the transcript expression per CML cell and may provide additional information about the dynamics of *BCR::ABL1* transcriptional activity under ongoing TKI therapy.

We analyzed the *BCR::ABL1* MR kinetic in a total of 762 samples from 64 patients for which both DNA and RNA measurements were available. In general, copy numbers of *BCR::ABL1* transcripts and fusion genes correlated well (Spearman correlation coefficient r = 0.93 [*P* < 0.0001] (Fig. S2). However, in 35 samples (4.6%) DNA-based measurements were positive while RNA was negative, contrary 8 samples (1%) were RNA positive but DNA negative. These discrepancies are mainly due to preclinical factors such as delayed sample processing (RNA negative but DNA positive) or reduced sample quality and quantity.

**Figure 4:**
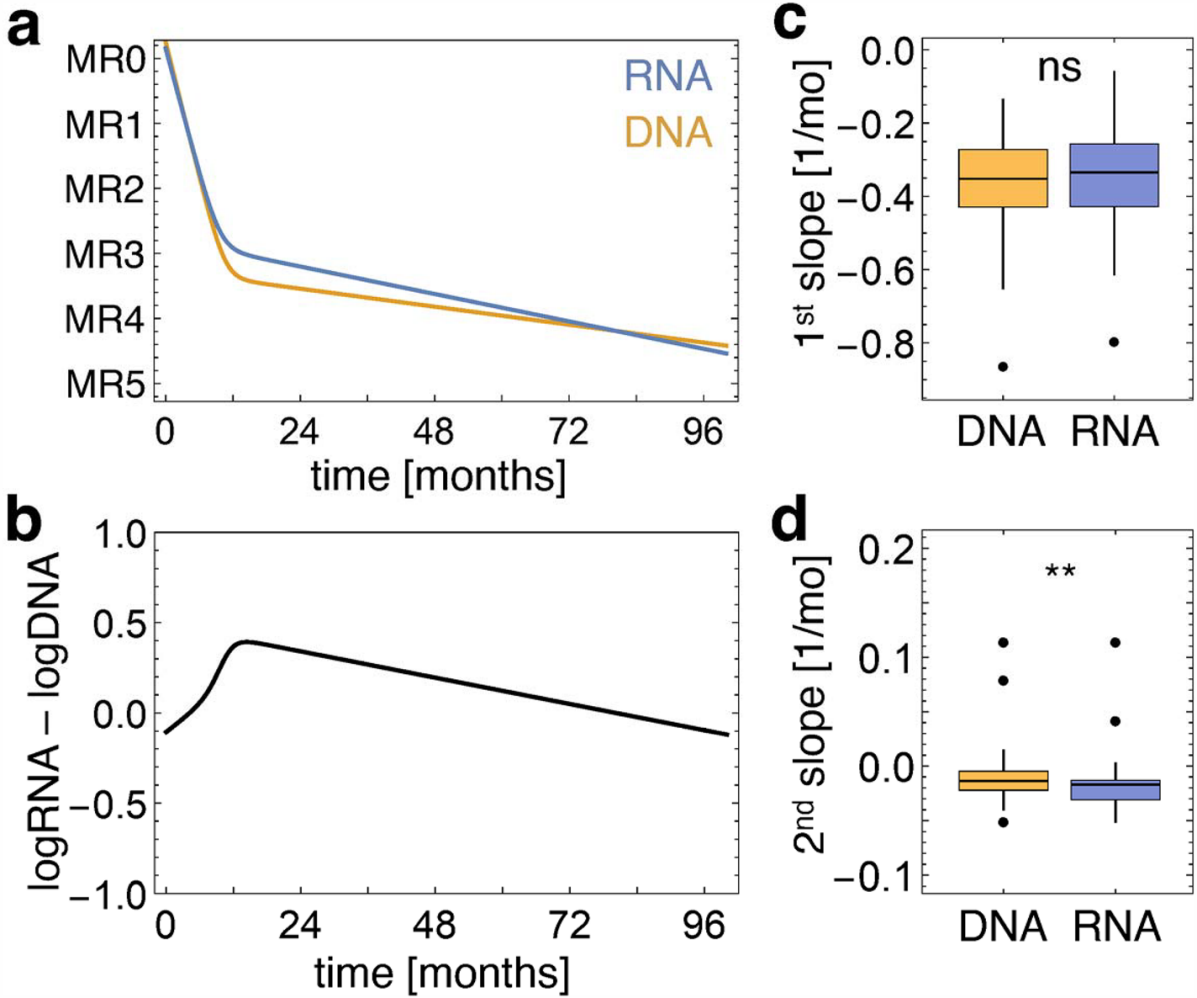
Bi-exponential modeling for the comparison of *BCR::ABL1* transcript levels and fusion gene numbers during TKI treatment over 5 years. (**a**) Comparison of RNA (blue) and DNA (orange) molecular response kinetics plotted over 96 months of therapy as bi-exponential graphs based on a population-based non-linear mixed effect model. (**b**) Difference of logarithmic transcript and fusion gene level (log10 RNA–log10 DNA) over 96 months adapted from the modeled bi-exponential kinetics for RNA- and DNA-based molecular response. (**c**) Slope α and slope β (**d**) of the RNA bi-exponential graph (blue) compared to the DNA bi-exponential graph (orange). ns not significant, **p < 0.01 (paired t-test).

For a quantitative comparison we fitted a bi-exponential model to both *BCR::ABL1* transcripts and fusion gene copy numbers using a non-linear mixed effect model. The modeled mean curves of RNA and DNA levels are provided in Fig. 4a over a period of 96 months after therapy start. They show similar kinetics with a rapid initial decrease (slope α, 1st slope) followed by a slower decrease (slope β, 2nd slope). The DNA curve shows a slightly steeper first decline and reaches a lower MR at the junction into the flatter section with MR3.3 at month 12.3 compared to MR2.9 at month 11.5 in the RNA curve. In the second, flatter section, the RNA curve has a steeper kinetic, so the graphs converge and intersect at month 80. The differences between RNA and DNA in the steepness of the slope are plotted for the first slope (α slope) (*p* = 0.31) in Fig. 4c and the second slope (β slope) (*p* = 0.0012) in Fig. 4d.

Figure 4b illustrates the ratio of transcript level to fusion gene level (log10 RNA–log10 DNA) based on the estimated RNA and DNA dynamics of the bi-exponential model shown in Fig. 4a. After an initial increase of the normalized *BCR::ABL1* gene expression, we observe a continuous slow decline after the maximum at about month 12, implying the transcription activity decrease.

## Discussion

Based on the German pediatric CML cohort we have established a framework to quantitatively monitor and continuously evaluate molecular treatment response in pediatric CML patients that complements current ELN guidelines. ELN-defined milestones have been validated for the prediction of outcome in CML in adult patients and are currently being adopted for pediatric CML, although it is known that the initial presentation and the therapy response show age-dependent differences^20,35,36^. Thus, in the present cohort initial leukocyte levels, which are often highly elevated in pediatric CML patients, show a significant correlation with the achievement of milestones at months 3 and 6.

Therefore, we evaluated the course of treatment response to imatinib in children and adolescents in a descriptive manner to represent real-life data and to conclude the extent to which consistent treatment adjustment is performed. Based on a subset of *optimal responders* we derived an optimal response target curve to which individual measurements at all time points can be compared. This continuous quantile format is the essential novelty of our approach as it allows to compare a patient-specific measurement at any time point to a suitable reference and to categorize the *BCR::ABL1* data as optimal, intermediate, and poor MR apart from only assessing the typical milestones at months 3, 6, and 12.

In our cohort, only approximately half of the patients presented with an optimal response. This may be due to a suboptimal therapy adjustment caused by non-optimal imatinib levels or the presence of variants affecting genes involved in drug absorption, distribution, metabolism, and excretion^37,38^. Furthermore, compliance is a major issue in children and adolescents^12,39^. A graphical representation as a feedback tool can support patient education in cases of insufficient adherence to therapy and potentially contribute to a more stringent adherence, which may increase the number of patients in DMR and TFR.

Rapid achievement of DMR offers the possibility to consider early TKI discontinuation to minimize adverse effects such as longitudinal body height stunting. Achieving an early major molecular response (equivalent to MR3) and DMR as soon as possible is associated with a better long-term prognosis, a reduced risk of disease progression to advanced stages, an increased chance of reaching TFR^19,40,41^, and can reduce long-term side effects and treatment costs.

Parallel quantification of *BCR::ABL1* genomic fusion gene sequences and fusion transcripts has already proven valuable for assessing DMR and the chance of sustained TFR^25,33,34^. A comparative analysis of *BCR::ABL1* transcript and fusion gene copy numbers in 64 pediatric CML patients where sufficient material was available for additional DNA-based quantification, revealed overall comparable kinetics for both molecular markers during imatinib therapy. Slight differences in the RNA and DNA-based course of our bi-exponential model show that during the first 5–6 months after treatment start, DNA levels are higher compared to RNA levels. Thereafter the relationship reverses, which is in line with published data from adult CML patients^42^ and indicates changes in the *BCR::ABL1* expression per CML cell. With longer treatment duration, the *BCR::ABL1* transcript quantity decreases gradually supporting the biological hypothesis of non-transcript producing quiescent CML cells also in pediatric CML patients^43,44^. It must be taken into account that in our mathematical analysis, the outcome later in the course of TKI therapy may be affected by the higher stability of DNA for pre-analytics, the potential variation in gene expression of the control gene, and different sensitivities in some of the individual DNA PCR assays. To minimize this effect, we set MR4.5 as the detection limit for both DNA and RNA values. The defined period for sample inclusion can also influence the mathematical estimation of the bi-exponential model. However, the parallel analyses of two different scenarios, 5 years follow-up (Fig. 4) and 6 years follow-up (Fig. S3), respectively, show comparable results.

Although higher patient numbers increase overall confidence, we believe limiting the number of cases to uniform and well-monitored cases is a preferable strategy, especially for establishing a reference. Therefore, we include in our analyses only pediatric CML patients diagnosed in the chronic phase included in the German CML-PAED-II trial and the subsequent registry. Nevertheless, the total number of measurement time points analyzed can be considered comparatively large for pediatric CML. Due to the low incidence of childhood CML, increased numbers of patients can only be obtained from international patient cohorts, which in turn, adds additional variability to the measurements. The retrospective evaluation of treatment data collected over a long time is a limitation of our analysis. Hence, our moving quantiles approach is based only on data from patients treated with imatinib as the first-line therapy.

The approach described here can be implemented in an analogous way for adults and offers additional options for application due to the larger patient numbers and thus higher statistical power. Basically, the continuous thresholds defined by moving quantiles have the advantage of enabling objective evaluation of measurements also between and after the well-established milestones. This additional information plays a particular role in two therapy phases: in the first months after diagnosis (up to month 6) and after reaching MMR or the milestone at month 12. In the early therapy phase, the moving quantiles allow to classify the results of additional, narrower time points with regard to the expected subsequent course and thus to support the decision for an early switch of therapy in case of a non-optimal response by several measurement time points. After reaching MMR or after the milestone at month 12, assessment of the monitoring results over time reveals the dynamics of the decline range between MR3 and MR4.5 and thus potentially an additional parameter for estimating the chances of TFR. These hypothetical considerations obviously need to be tested and validated in prospective studies.

In summary, we calculated for the first time continuous therapy response references specific for pediatric CML treatment response assessment based on *BCR::ABL1* transcript levels. This is particularly important in children to monitor compliance, or to assist with temporary or permanent TKI cessations for reduction of side effects e.g. on growth inhibition. The methodology used here can also be applied to the continuous representation of any other biomarker for therapy monitoring and could thus easily be adapted to patients with initial 2G-TKI treatment or adult CML patients.

### Supplementary Information

Supplementary Figures.

## Data Availability

The datasets used and analyzed during the current study are available from the corresponding author on reasonable request.
