# Supplementary material for: Continuous therapy response references for BCR::ABL1 monitoring in pediatric chronic myeloid leukemia
Source: Sci Rep. 2023 Oct 24;13:18199. doi: 10.1038/s41598-023-45364-0 (PMC10598002; doi:10.1038/s41598-023-45364-0)
Supplement: Supplementary file 1 — Supplementary Figures. [file 41598_2023_45364_MOESM1_ESM.pdf]

Supplementary Figures

Figure S1

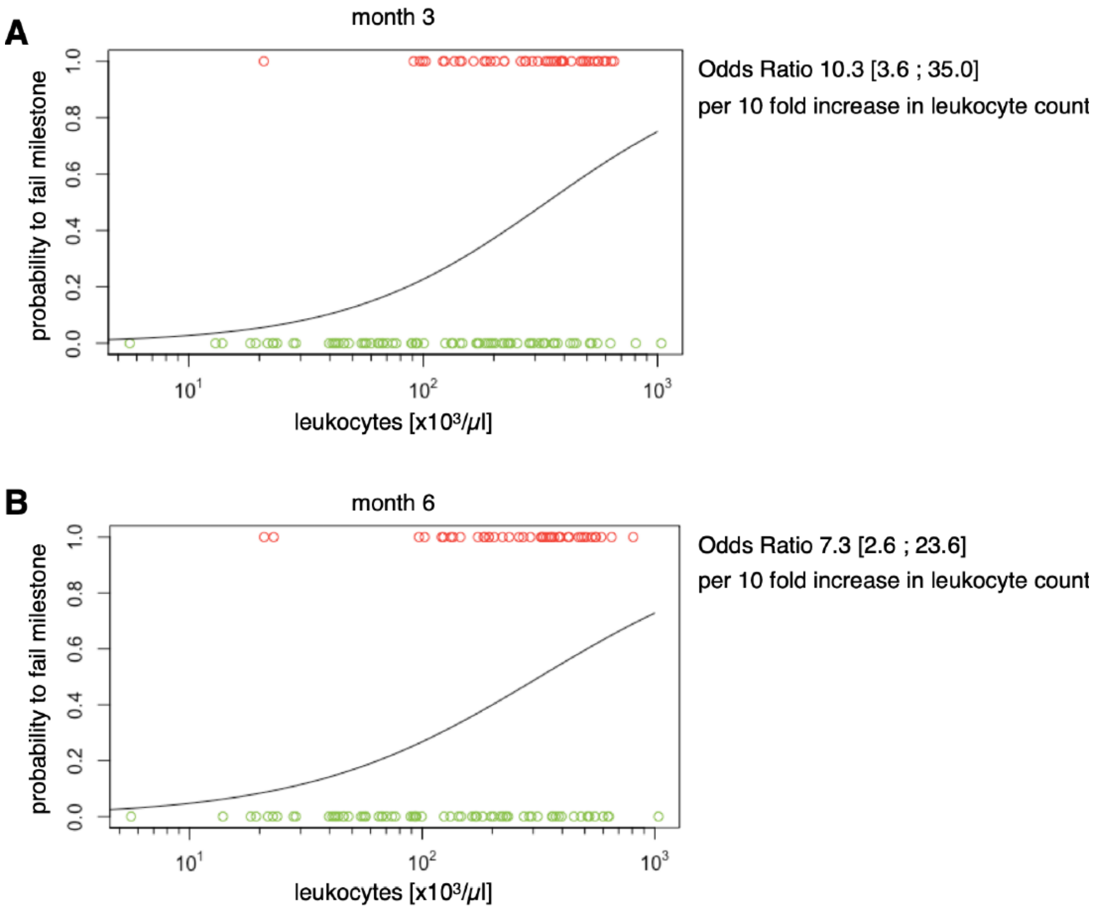

Figure S1. Logistic Regression to estimate risk of failing milestones at month 3 (A) and month 6 (B). Leukocyte counts are depicted on a logarithmic scale in green for patients that reach the respective milestone and in red for patients that fail them. Black curves represent the estimated probability to fail the milestone. Logistic Regression was performed on logarithmic leukocyte counts and result in odd ratios of 10.3 (95% confidence interval [3.6 ; 35.]) and 7.3 [2.6 ; 23.6] resp., for a 10 fold increase in leukocyte count.

Figure S2

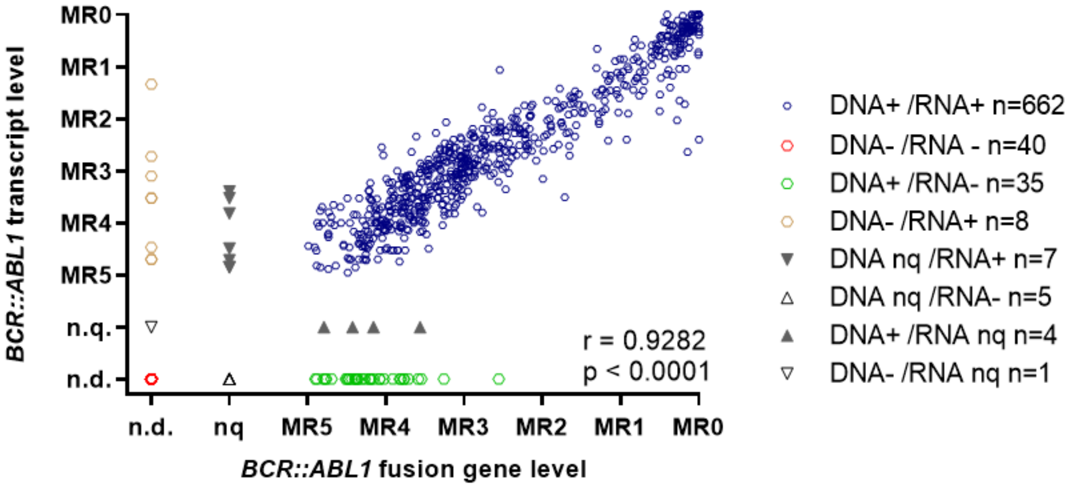

Figure S2. Correlation of the 762 *BCR::ABL1* fusion genes and transcripts measured in parallel in 64 pediatric CML patients.
n.d. not detectable, n.q. not quantifiable

Figure S3

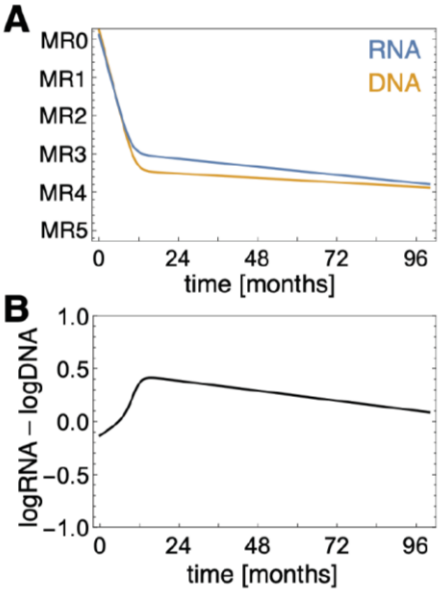

Figure S3. Biexponential modelling for the comparison of *BCR::ABL1* transcript and fusion gene numbers during TKI treatment over 6 years. (A) Comparison of RNA (blue) and DNA (orange) molecular response kinetics plotted over 96 months of therapy as biexponential graphs based on a population-based non-linear mixed effect model. (B) Difference of logarithmic transcript and fusion gene level (log10 RNA – log10 DNA) over 96 months adapted from the modeled biexponential kinetics for RNA and DNA based molecular response.
